# In-vitro compatibility assay of indigenous *Trichoderma* and *Pseudomonas* species and their antagonistic activities against black root rot disease (*Fusarium solani*) of faba bean (*Vicia faba* L.)

**DOI:** 10.1186/s12866-021-02181-7

**Published:** 2021-04-17

**Authors:** Alemayehu Dugassa, Tesfaye Alemu, Yitbarek Woldehawariat

**Affiliations:** 1 Department of Microbial, Cellular and Molecular Biology, College of Natural and Computational Sciences, Addis Ababa University, Addis Ababa, Ethiopia; 2 Department of Zoological Science, College of Natural and Computational Sciences, Addis Ababa University, Addis Ababa, Ethiopia

**Keywords:** Compatibility, Fungicides, Pathogenicity, *Pseudomonas*, Black root rot, *Trichoderma*, *Vicia faba* L

## Abstract

**Background:**

Faba bean *(Vicia faba* L.) cultivation is highly challenged by faba bean black root rot disease *(Fusarium solani)* in high lands of Ethiopia. To ensure sustainable production of faba beans, searching for eco-friendly disease management options is necessary to curb the progress of the disease timely. The indigenous biocontrol agents that suit local environments may effectively strive with in-situ microorganisms and suppress local pathogen strains. This study aimed to screen antagonistic indigenous compatible *Trichoderma* and *Pseudomonas* strains against *Fusarium solani.* In the pathogenicity test, soil-filled pots were arranged in complete random block design and sown with health faba bean seeds. The effect of some fungicides was evaluated against *Fusarium* by food poisoning methods to compare with the biocontrol agents*.* The antagonistic efficacy of biocontrol agents and their compatibility was investigated on Potato dextrose agar medium.

**Results:**

*Fusarium solani* AAUF51 strain caused an intense root rotting in faba bean plant. The effect of Mancozeb 80% WP at 300 ppm was comparable with *Trichoderma* and *Pseudomonas* strains against *Fusarium.* The mycelial growth of test the pathogen was significantly (*P* ≤ 0.05) reduced to 86.67 and 85.19% by *Trichoderma harzianum* AAUW1 and *Trichoderma viridae* AAUC22 strains in dual culture, respectively. The volatile metabolites of *Pseudomonas aeruginosa* AAUS31 (77.78%) found the most efficient in reducing mycelial growth of *Fusarium* followed by *Pseudomonas fluorescens* AAUPF62 (71.11%) strains. The cell-free culture filtrates of *Pseudomonas fluorescens* AAUPF62 and *Pseudomonas aeruginosa* AAUS31 were more efficient than the *Trichoderma* strain in reducing the growth of *Fusarium* isolates. There was no zone of inhibition recorded between *Trichoderma harzianum* AAUW1*, Trichoderma viridae* AAUC22*, Pseudomonas aeruginosa* AAUS31*,* and *Pseudomonas fluorescens* AAUPF62 strains, hence they were mutually compatible.

**Conclusions:**

The compatible *Trichoderma* and *Pseudomonas* strains showed antagonistic potentiality that could be explored for faba bean protection against black root rot disease and might have a future dual application as biocontrol agents.

## Background

Faba bean (*Vicia faba* L.) belongs to the family Fabaceae is a pulse crop grown in many regions in the world due to its high nutritional value [1] and effective biological nitrogen fixation [2]. Nutritionally, it is rich in protein, complex carbohydrates, dietary fiber, choline, lecithin, minerals, and secondary metabolites including phenolic compounds [3]. Also, faba bean seeds are low in fats, cholesterol-free, and low sodium [4]. Hence, it is globally recognized as one of the most important staple crops for human consumption and essential to attaining sustainable food security [2].

Faba bean takes the largest share of area (437,106.04 ha) and production (9,217,615.35 quintals) among pulses grown in Ethiopia [5]. Despite its wide ecological and economic benefits, the average actual yield of faba bean is about 1.2 t ha^− 1^ which is very low compared to the average potential yield (4.4 t ha^− 1^) in major production areas of the country [6]. The low yield of faba bean is attributed to both biotic [7] and abiotic [8] constraints.

Fungal diseases are among the most important biotic factors causing faba bean yield reduction [2]. Black root rot caused by *Fusarium solani* is the most destructive and faba bean yield-reducing among fungal diseases in the high lands of Ethiopia [9]. The disease impacting the production of faba beans and causes up to 100% yield loss on farmers’ fields under severe conditions [10]. It usually appears at pre-emergence and early seedling stages during the growing season [2]. It is noted by stunted plant growth and yellowing, necrotic basal leaves, brown, red, or black streaks on roots that coalesce as they mature. The disease causes rotting which makes black discoloration of the roots and ultimately results in the death of the plant [1].

Although black root rot disease has been known as a common disease of faba bean for a long time, management options for faba bean producers are limited. To maintain the level of faba bean yield both quantitatively and qualitatively, farmers often rely heavily on the use of chemical fungicides to control the disease [11]. Conversely, the application of chemical fungicides in faba bean growing field results in the development of resistance by the plant pathogens, hazardous to human health, pollute the environment and harm biodiversity of the ecosystem [12].

Thus, the use of supplementary biological control methods is mandatory to suppress the effect of black root rot diseases and to ensure sustainable eco-friendly agricultural practice. A biological control method becomes a promising tool to use in agricultural production as it reduces the release of polluting chemical fungicides to the ecosystem [13].

At present, quite several biocontrol agents are found to be able to manage plant diseases effectively as well as all ecologically soundproof [14]. *Trichoderma* and *Pseudomonas* species have been reported as the most potential biocontrol agents against numerous phytopathogens [15]. The plant growth-promoting *Pseudomonas aeruginosa* have increased the yield by suppressing the disease effect [16]. However, the use of a single biocontrol agent often resulted in inconsistent performances in agriculture [17].

One of the reasons for such a failure could be that a single bioagent might not grow equally well in a variety of environmental conditions [18]. Nowadays, more emphasis was laid on the use of combined biocontrol agents with multiple mechanisms that could increase the success of biological control [19]. To designate microbial for combined use, the approach was focused on the compatibility among selected bioagents [20]. Co-inoculation of compatible biocontrol agents is considered to be an innovative approach in plant health management, and for the improvement of crop yield and quality. Therefore, the current study was aimed to identify compatible indigenous *Trichoderma* and *Pseudomonas* strains which showed antagonistic activities against black root rot disease (*Fusarium solani*) of Faba bean (*Vicia faba* L.).

## Materials and methods

### Fungal pathogen

#### Sampling techniques and sample collection

A total of 100 naturally infected faba bean roots with black root rot symptoms were collected randomly from North Shoa farmers’ fields of Angolelana Tera (50 samples), and Sululta (50 samples) districts during the 2018 main cropping season. All samples were packed in polyethylene bags and transported to the Mycology Laboratory, College of Natural and Computational Sciences, Addis Ababa University. The samples were stored at 4 °C until use.

#### Samples preparation for isolation, purification, and identification of *Fusarium solani*

According to [21], the infected parts of the root samples were washed in running tap water and excised with a sterile scalpel to small pieces (1 cm). The pieces were surface-sterilized by dipping those in 2% sodium hypochlorite for 5 min, followed by washing three times with sterile distilled water for 2 min. The fragments were inoculated on 90 mm Petri plates of sterilized Potato Dextrose Agar (PDA) medium amended with chloramphenicol. A 0.1 ml of the last washes was spread plated on PDA as a control to check the quality of sterilization. The inoculated medium was incubated at 25 ± 2 °C for 7 days. Triplicates were maintained for each treatment. The colonies were purified using single hyphal tip techniques followed by [22] and identified based upon their cultural and morphological characteristics. The spore shape of the isolate was observed under high power objectives (40X) lens by staining the pure colony with lactophenol cotton blue.

#### Inoculum preparation of test pathogen

Four mm discs of five selected faba bean black root rot diseases causal agent suspected isolates were removed by using sterilized cork borers. Three discs of each isolate were separately inoculated in a 250 ml flask containing 100 ml Potato dextrose broth and incubated at 25 ± 2 °C for seven days. The levels of spore suspensions were adjusted to 1 × 10^6^ spores ml^− 1^ by using a hemocytometer [23].

#### Pathogenicity tests

In this experiment, the seed of susceptible faba bean cultivar (FB-26869) were collected from the Ethiopian Biodiversity Institute (EBI) and used to carry out the pathogenicity tests of *Fusarium* strains. The pathogenicity tests were carried out under greenhouse conditions located at the College of Natural and Computational Sciences, Addis Ababa University. An autoclave sterilized mixture of 1:1 clay soil and sand were distributed in plastic pots (2 kg pot^− 1^). The pots were kept under 12 h light and 12 h dark photoperiod and watered every day to maintain the moisture of the soil. The mean minimum and maximum daily temperature in the greenhouse during the study period were 15 °C and 22 °C, respectively. Likewise, the mean relative humidity was between 65 and 85%.

Surface sterilized health faba bean seeds were sown in each pot (4 seeds pot^− 1^). Ten ml of spore suspension (1 × 10^6^ spores ml^− 1^) was poured onto the stem base of each faba bean plant separately a week after sowing followed by [23]. Pots without the test pathogen used as control. Triplicates were maintained for each treatment. Faba bean plants were examined periodically and plants with black root rot symptoms were recorded to determine the virulence of each pathogen under test. Pathogenic *Fusarium* was re-isolated from infected plants and re-inoculated as mentioned above. Data were recorded as the percentage of disease incidence and severity index in each treatment.

$$ \mathrm{Percent}\ \mathrm{disease}\ \mathrm{incidence}=\frac{\mathrm{No}.\mathrm{of}\ \mathrm{disease}\mathrm{d}\ \mathrm{plants}\ }{\mathrm{Total}\ \mathrm{no}.\mathrm{of}\ \mathrm{plants}\ \mathrm{inspected}}\times 100 $$

Disease severity ratings were conducted using a 0–5 rating scale for overall plant and root symptoms as follows: The overall plant rating was as follows: 0 = no symptoms, 1 = minor plant stunting, 2 = wilting or drooping of a few leaves, 3 = plant stunting obvious, wilting leaves with chlorosis and some necrosis, 4 = extreme plant stunting and shoot showing external brown discoloration, and 5 = death of the plant. After 60 days, faba bean plants from each treatment were uprooted, washed in running water, and air-dried. The mean disease rating of the root is computed as the disease severity index. Roots were rated as follows: 0 = no symptoms, 1 = minor root tip discoloration, 2 = root discoloration extending beyond root tips, 3 = prominent dark root discoloration, smaller roots more severely affected. 4 = smaller, secondary roots are black or missing, 5 = only tertiary roots remaining with brown/black coloration. The disease severity index (DSI) was computed for each plant by adding the individual ratings comprising overall plant and root ratings [24].

$$ \%\mathrm{DSI}=\left\{\frac{\ \left[\sum \Big(\mathrm{Number}\ \mathrm{of}\ \mathrm{plants}\ \mathrm{showing}\ \mathrm{infected}\ \mathrm{roots}\ \mathrm{X}\ \mathrm{disease}\ \mathrm{severity}\right)\Big]}{\left(\mathrm{Number}\ \mathrm{of}\ \mathrm{total}\ \mathrm{plants}\ \mathrm{X}\ \mathrm{the}\ \mathrm{hieghest}\ \mathrm{disease}\ \mathrm{severity}\right)}\right\}X\ 100 $$

#### Sensitivity of *Fusarium* isolate to fungicides

The sensitivity of Fusarium isolates to some fungicides was evaluated under in vitro conditions. Two fungicides with different active ingredients; Mancozeb 80% WP and Cupper Oxy-Chloride 50% WP were collected from the Addis Ababa market and used as comparing treatment. For each fungicide, a stock solution having a concentration of 1000 ppm was prepared followed by commercial formulation [25]. The calculated amount of the stock solution of a fungicide was supplemented with sterilized PDA medium and chloramphenicol to get the final concentrations of 50,100, 200, 300, and 400 ppm in food poisoning techniques. Twenty ml of the supplemented medium of a particular concentration was poured in sterilized Petri plates and allowed to solidify. In the control set, the required amount of sterile water instead of fungicide was added to the PDA medium. Then it was inoculated in the center of the plate with a 5 mm of mycelial agar disc of Fusarium isolate and incubated at 25 ± 2 °C for seven days. Three replications were maintained in both cases.

### Collection of *Pseudomonas* and *Trichoderma* strains

Five *Pseudomonas* strains (*P. aeruginosa* AAUAm28, *P. fluorescens* AAUF3, *P. fluorescens* AAUW24, *P. aeruginosa* AAUS31, *P. fluorescens* AAUPF62) and five *Trichoderma* strains (*T. harzianum* AAUW1, *T. viridae* AAUC22, *T. hamatum* AAUS69, *T. hamatum* AAUS12, *T. reesei* AAUAm31) were obtained from the stock cultures in the Mycology Laboratory, Department of Microbial Cellular and Molecular Biology, College of Natural and Computational Sciences, Addis Ababa University, Ethiopia. These strains were originally isolated from health faba bean rhizosphere soil from different localities of Angolelana Tera, Sululta, and Medakegn districts. The Potato Dextrose Agar (PDA) medium supplemented with chloramphenicol was used for reactivation, multiplication, and preservation of *Trichoderma* strains while Nutrient agar medium was used for *Pseudomonas* strains.

### In-vitro antagonistic assay of *Trichoderma* and *Pseudomonas* strains

#### Dual culture antagonistic assay

In dual culture, an agar disc (4 mm) of seven days old *Trichoderma* was placed 10 mm away from the periphery on PDA plates and the same sized agar disc of seven days old test pathogen was placed at the opposite side of *Trichoderma* isolate. The bacterial isolate was streaked at the opposite side of the test pathogen; approximately 40 mm away from the center followed by [26]. A medium inoculated only with *Fusarium solani* was used as a control and triplicates were maintained. The plates were incubated at 25 ± 2 °C until full growth of the control. After the period of incubation, the percentage of inhibition of the test pathogen was calculated as described by [27].

$$ \% inhibition=\frac{\left(R1-R2\right)\times 100}{R1} $$

where; R1: mycelial growth in control, R2: mycelial growth in dual culture.

#### The effect of antifungal volatile organic metabolites

The effect of volatile organic metabolites of both *Trichoderma* and *Pseudomonas* isolates on the mycelial growth of the test pathogen was tested by paired plate techniques followed by [28]. A Petri plate containing PDA was separately inoculated with a 4 mm agar plug of activated test pathogen and *Trichoderma* isolates at the center of the plate. Whereas a Petri plate containing Nutrient Agar medium was streak inoculated with a loopful of 48 h old bacterial isolate followed by [26]. Half plates with the test pathogen inverted over a plate containing *Trichoderma*, and bacterial isolates. Both half plates were sealed together by scotch tape and the paired plates were incubated at 26 ± 2 °C for 14 days. A Control set of paired plates was designed with only the test fungus on the PDA half-plate inverted over the uninoculated medium. The experiment was conducted in triplicates. After the incubation period, the paired plates were observed for inhibition of the mycelial growth of the test pathogen, and percent inhibition of the test pathogen was calculated as mentioned above.

#### The antagonistic assay of culture filtrates of *Trichoderma* and *Pseudomonas* isolates

A 250 ml flask containing 100 ml of potato dextrose broth (PDB) was separately inoculated with five days old culture of three an equal disc (4 mm) of each *Trichoderma* isolate and incubated at 25 ± 2 °C on a rotary shaker set at 100 rpm for 14 days. The culture was filtered using filter paper (Whatman No.1) for removing mycelial mats. Then, the filtrate was sterilized by passing through a 0.2 μm pore biological membrane filter [20]. Also, the Pseudomonas was inoculated into a 250 ml conical flask containing 100 ml of nutrient broth. The flasks were incubated at 28 ± 2 °C for 96 h on an orbital shaker with 120 rpm. The culture was centrifuged at 10,000 rpm for 30 min to get the cell-free filtrate [22].

Each sterilized filtrate was mixed to molten PDA medium amended with chloramphenicol at 40 ± 3 °C to obtain a final concentration of 15, 25, and 40% (v/v) into a sterilized 250 ml flask. Finally, about 20 ml of the medium amended with different concentrations of the filtrate were poured in 90 mm Petri plates. The secondary metabolites in the filtrate were tested for their efficacy against the test pathogens. The test pathogen was centrally inoculated with an individual equal disc (4 mm) of seven days old culture. PDA plates inoculated with pathogens without culture filtrates served as control. Three replicates were maintained for each treatment and incubated at 25 ± 2 °C. The percentage inhibition of mycelial growth was calculated as mentioned above.

### Test for compatibility of *Trichoderma* and *Pseudomonas* isolates

A dual culture technique was performed for evaluating the compatibility of the isolates [29]. *Trichoderma* grown on PDA plates at 25 °C for 5 days, were used for the study. *Pseudomonas* isolates were raised in Nutrient broth. Twenty-four hrs old bacterial cultures were streaked on one side at 10 mm away from the periphery of the Nutrient Agar plates. The bacterial isolates were allowed to grow for 24 h. at 26 ± 2 °C. A four mm diameter plug from a five days old culture of *Trichoderma* isolate was placed in the opposite direction of the plate (approximately 40 mm apart). A control plate was kept without bacterial inoculation. Triplicates were maintained for each treatment. After five days of incubation at 26 ± 2 °C, the growth of *Trichoderma* isolates in the dual inoculation plates was measured for assessing compatibility. The zone of inhibition was measured and percent inhibition over control was calculated using the above-mentioned formula.

#### Data analysis

Data were subjected to SPSS statistical software version 20 and one-way ANOVA. The effect of antagonists on the mycelial growth of the test pathogen was compared using the least significant difference (LSD) at a 5% probability level (*P* ≤ 0.05).

## Results

A total of 25 *Fusarium* isolates were obtained from infected faba bean roots. However, five fungal isolates which exhibited the basic characteristics feature of *Fusarium solani* were selected and designated as showed in Table 1.

**Table 1:** Localities and designation *of Fusarium solani* strains

Zone	Districts	Locality	Designated as	Identified as
North Shoa	Sululta	Duber	AAUF51	*Fusarium solani* AAUF51
North Shoa	Angolelana Tera	Sariti	AAUF52	*Fusarium solani* AAUF52
North Shoa	Sululta	Wakene	AAUW22	*Fusarium solani* AAUW22
North Shoa	Sululta	Wakene	AAUW41	*Fusarium solani* AAUW41
North Shoa	Sululta	Chagen	AAUC31	*Fusarium solani* AAUC21

### Cultural and morphological characters of *Fusarium* strains

**Fig. 1:**
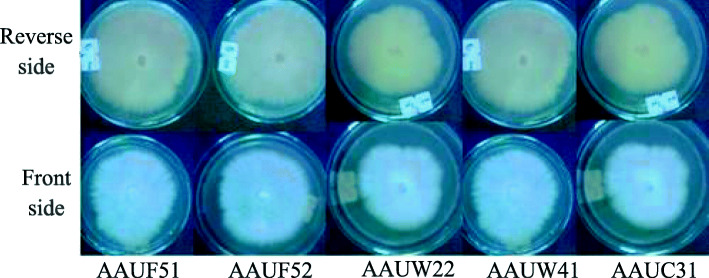
Colony morphology of *Fusarium solani* isolates

Five *Fusarium* isolates, *Fusarium solani* AAUF 51, AAUF52, AAUW22, AAUW41, and AUUC31 growing on a PDA had developed flat colony growth with pale coloration on the reverse side. All isolates had abundant white cottony growth on the front side. The colony margin of *Fusarium solani* AAUF51, AAUF52, and AAUW41 appeared smooth, whereas appeared irregular in *Fusarium solani* AAUW 22 and *Fusarium solani* AAUC31 isolates. In older cultures, some black bodies are scattered on the culture plate. Macroconidia were straight to slightly curve with thin walls. The apical cells were slightly curved and tapered. Chlamydospores of isolates were smooth, globose, usually single, light yellow-brown color with thick walls, immersed in the culture media rather than superficial. Significantly (*P* ≤ 0.05) higher colony diameter (90 mm) was recorded in isolates coded as *Fusarium solani* AAUF 52, AAUF 51, and AAUW 41 than AAUC 31 and AAUW 22 (Fig. 1).

### Pathogenicity of *Fusarium* isolates

**Fig. 2:**
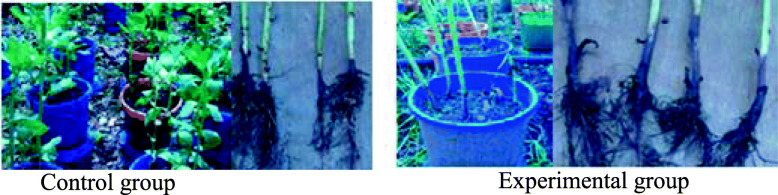
Pathogenicity test under greenhouse conditions

In the pathogenicity test, water-inoculated healthy faba bean plants showed no stunting. The root tissue was white with no internal or external discoloration and a 5% disease incidence. In contrast, *Fusarium*-infected faba bean plants initially showed mild stunting 3–4 weeks after inoculation, followed by more severe stunting later with 100% disease incidence. Roots were dark brown to black, severely decayed, weak, and easily separated from the surrounding soil (Fig. 2). Plants that were necrotic had dark brown external discoloration at the base of the stem.

The maximum mean percent disease severity index (DSI) was recorded by *Fusarium solani* AAUF51 strain (89.5%) followed by AAUF52 (89%), AAUW41 (88.5%), AAUW22 (86.4%), and AAUC21 (85%) (Fig. 2). The ANOVA results indicated that there was no significant difference (*P* > 0.05) between AAUF51, AAUF52, and AAUW41 isolates in virulence as determined by DSI on faba bean plants. Hence, due to the vastness of the data, we provide here only the antagonistic effect of bioagents against *Fusarium solani* AAUF51. The fungus was re-isolated from the diseased stem and root tissue of inoculated plants but not from healthy check plants. Symptoms on inoculated plants were similar to those observed in the field and the recovered isolates had the same cultural and morphological characteristics as the original isolates, thus fulfilling Koch’s postulates.

### Sensitivity of *Fusarium* isolate to fungicides

**Fig. 3:**
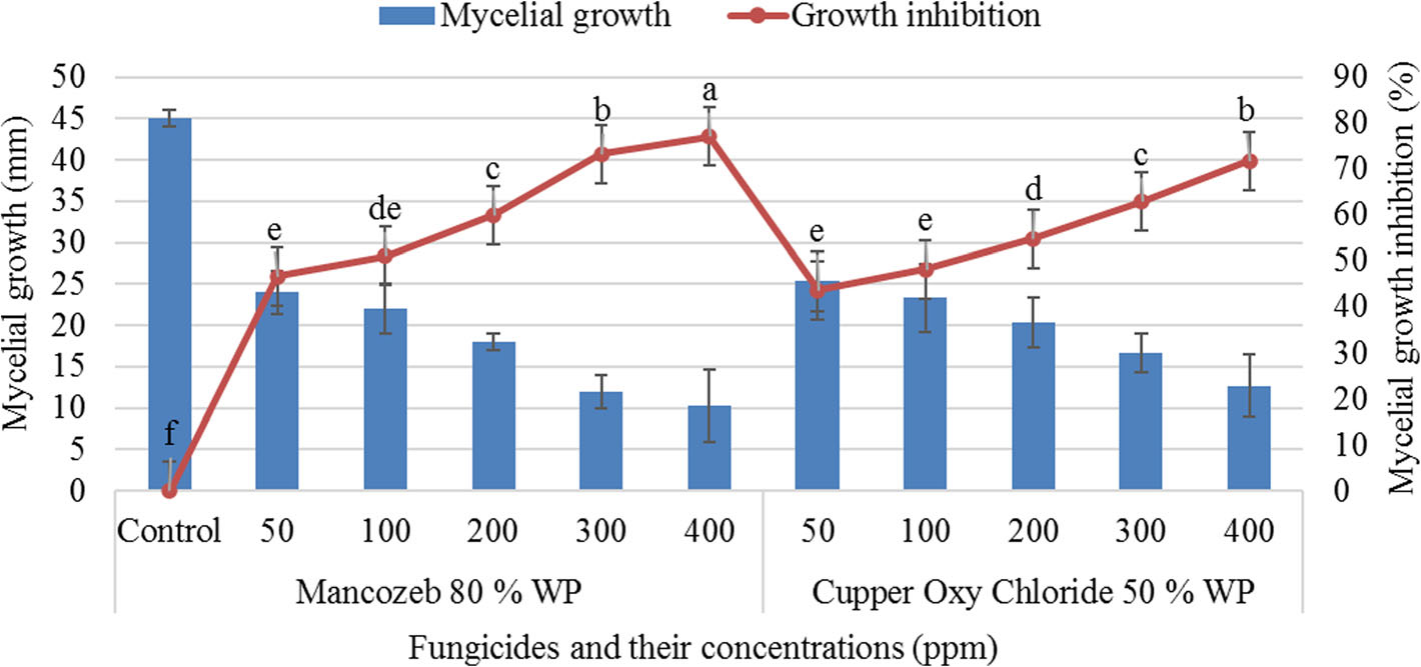
The effect of fungicides on mycelial growth of *Fusarium solani* AAU F51 strain

Better inhibition of the radial growth of the test pathogen was observed with Mancozeb 80% WP at 50, 100, 200, 300, and 400 ppm concentration than Cupper Oxy-Chloride 50% WP after seventh days of incubation. Mancozeb 80% WP was responsible for more than 71% inhibition of *Fusarium* isolates at 300 whereas Cupper Oxy-Chloride 50% WP was responsible at 400 ppm concentrations. There was no significant difference (*P* > 0.05) among 300 Mancozeb 80% WP and 400 ppm Cupper Oxy-Chloride 50% WP concentration in percent inhibition of the radial growth of the test pathogen. Hence, *Fusarium* isolate was more sensitive to Mancozeb 80% WP than Cupper Oxy-Chloride 50% WP even at low concentrations (Fig. 3).

A vertical line indicates a standard error; the same letter represents an absence of significant difference at *P* > 0.05.

### Antagonistic assay of *Trichoderma* and *Pseudomonas* isolates against the test pathogen

#### Dual culture antagonistic test

**Fig. 4:**
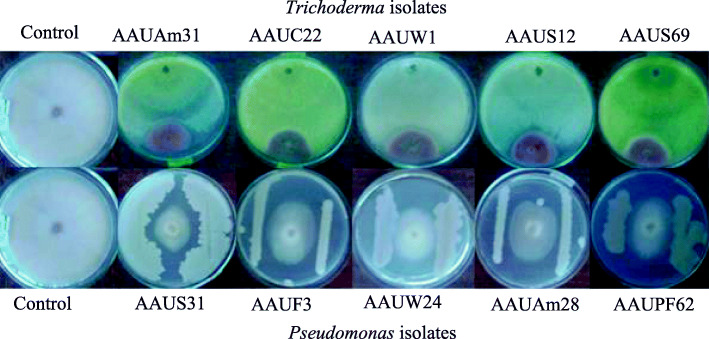
Dual culture of *Pseudomonas* and *Trichoderma* strains against *Fusarium solani* AAU F51

**Fig. 5:**
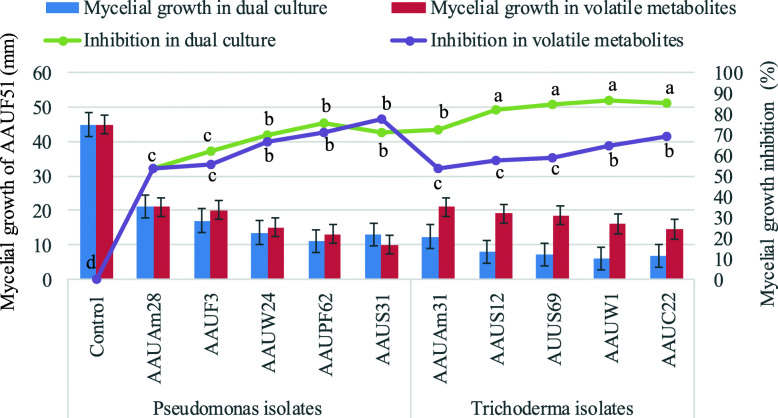
The effect of bioagents on mycelial growth of *Fusarium solani* AAUF 51 both in dual culture and volatile metabolites

The in-vitro dual culture test is one of the key tests used for the preliminary screening of biological control agents. Most of the *Trichoderma* and *Pseudomonas* isolates showed significant potent antifungal activity by restricting mycelial expansion. *Trichoderma viridae* AAUC22 (3.81 mm), *Trichoderma harzianum* AAUW1 (3.33 mm), *Trichoderma hamatum* AAUS12 (3.00 mm), *Trichoderma hamatum* AAUS69 (2.67 mm), and *Trichoderma reesei* AAUAm31 (2 mm) displayed zone of inhibition against *Fusarium solani* AAUF51 isolate. *Trichoderma* isolates showed a higher inhibition effect than *Pseudomonas* isolates. *Trichoderma harzianum* AAUW1 was the best bioagent followed by *Trichoderma viridae* AAUC22 inhibiting 86.67 and 85.19% mycelial growth of the test pathogen in dual culture, respectively. *Pseudomonas fluorescens* AAUPF62 and *Pseudomonas aeruginosa* AAUS31 isolates caused an inhibition area of 75.56 and 71.11%, respectively (Fig. 4 & Fig. 5).

#### Volatile antifungal metabolites

It is observed from this experiment that all the tested bioagents produced volatile antifungal metabolites having a significant effect in reducing the mycelial growth of *Fusarium solani*. *Pseudomonas aeruginosa* AAUS31 (77.78%) was found the most efficient in reducing the mycelial growth of *Fusarium solani* AAUF51. *Pseudomonas fluorescens* AAUPF62 exhibited significantly higher antagonistic volatile metabolites than others and inhibited the mycelial growth of test pathogen by 71.11% followed by *Trichoderma viridae* AAUC22 (67.78%) over the control. The volatile antifungal metabolites of *Pseudomonas fluorescens* AAUW24 and *Trichoderma harzianum* AAUW1 isolates had reduced the mycelial growth of *Fusarium* isolate to 66.67 and 64.44%, respectively. The least percent inhibition was recorded by *Pseudomonas aeruginosa* AAUAm28 (53.33%) and *Trichoderma* reesei AAUAm31 (53.33%) isolates (Fig. 5).

A vertical line indicates standard errors; the same letter represents an absence of significant difference at *P* > 0.05.

#### The effect of culture filtrates (non-volatile compound)

All tested bacteria and *Trichoderma* isolates significantly inhibited *Fusarium* isolates by the production of non-volatile antifungal secondary metabolites at 15, 25, and 40% concentrations of culture filtrates (Table 2). *Pseudomonas fluorescens* AAUPF62 was the most inhibiting for the test pathogen mycelial growth at the three culture filtrates 42.22, 60.00, and 82.22%, respectively. It is observed that culture filtrate from *Pseudomonas fluorescens* AAUPF62 (42.22%) and *Pseudomonas aeruginosa* AAUS31 (37.78%) isolates showed higher mycelial growth inhibition at a concentration of 15% than others. *Trichoderma viridae* AAUC22 has reduced the mycelia of the test pathogen by 33.33%, whereas *Trichoderma harzianum* AAUW1 reduced by 28.89%.

**Table 2:** The effect of culture filtrate of *Pseudomonas* and *Trichoderma* strains on mycelial growth of the test pathogen

Bioagents	Culture filtrate concentrations, and mycelial growth (mm), and growth inhibition (%)					
	15% filtrate		25% filtrate		40% filtrate	
	Mean ± SD	Inhibition (%)	Mean ± SD	Inhibition (%)	Mean ± SD	Inhibition (%)
*Pseudomonas* strains						
Control	45.00 ± 0.0	00.00^e^	45.00 ± 0.0	00.00^c^	45.00 ± 0.0	00.00^e^
AAUAm28	40.00 ± 2.0	11.11^d^	30.00 ± 1.5	33.33^b^	16.00 ± 1.5	64.44^c^
AAUF3	38.00 ± 0.8	15.56^c^	29.00 ± 2.1	35.56^b^	12.00 ± 0.8	73.33^b^
AAUW24	34.00 ± 1.0	24.44^b^	24.00 ± 2.0	46.67^b^	12.00 ± 0.7	73.33^b^
AAUS31	28.00 ± 3.0	37.78^a^	20.00 ± 2.0	55.56^a^	10.00 ± 4.0	77.78^a^
AAUPF62	26.00 ± 1.2	42.22^a^	18.00 ± 1.0	60.00^a^	8.00 ± 2.0	82.22^a^
*Trichoderma* strains						
AAUW1	32.00 ± 2.3	28.89^b^	22.00 ± 1.6	51.11^a^	12.00 ± 1.0	73.33^b^
AAUC22	30.00 ± 0.8	33.33^b^	20.00 ± 2.0	55.56^a^	11.00 ± 0.6	77.78^a^
AAUS69	36.00 ± 1.5	20.00^c^	26.00 ± 1.4	42.22^b^	18.00 ± 2.0	60.00^c^
AAUS12	39.00 ± 1.1	13.33^c^	28.00 ± 3.0	37.78^b^	20.00 ± 3.0	55.56^d^
AAUAm31	43.00 ± 2.3	4.44^d^	30.00 ± 1.2	33.33^b^	22.00 ± 0.8	51.11^d^

SD; Standard Deviation, the same letter within columns indicates absence of significant difference at P > 0.05

The maximum percent inhibition was observed in the culture filtrate of *Pseudomonas fluorescens* AAUPF62 (82.22%) at 40% concentration followed by *Pseudomonas aeruginosa* AAUS31 (77.78%) and *Trichoderma harzianum* AAUW1 (77.78%). The least mycelial growth inhibition of the test pathogen was recorded by *Trichoderma reesei* AAUAm31 (4.44%) at a 15% concentration of culture filtrate. Increasing the concentrations of culture filtrate caused an increase in growth inhibition (Table 2).

### In vitro compatibility test of *Trichoderma* and *Pseudomonas* strains

As data are shown in Table 3, fourteen out of 25 combinations between *Pseudomonas* and *Trichoderma* strains were incompatible. Antagonists grew normally in the control. *Trichoderma viridae* AUUC22 exhibited no antagonism against *Pseudomonas aeruginosa* AAUAm28, *Pseudomonas aeruginosa* AAUW24, *Pseudomonas aeruginosa* AAUS31, and *Pseudomonas fluorescens* AAUPF62. No zone of inhibition was found between *Pseudomonas fluorescens* AAUW24 and *Trichoderma harzianum* AAUW1, *Trichoderma viridae* AAUC22, *Trichoderma hamatum* AAUS69, and *Trichoderma hamatum* AAUS12 strains. Besides, no antagonism was recorded by *Trichoderma harzianum* AAUW1 against *Pseudomonas aeruginosa* AAUS31, and *Pseudomonas fluorescence* AAUPF62. Moreover, *Trichoderma viridae* AAUC22 hadn’t exhibited antagonism against *Pseudomonas aeruginosa* AAUS31, and *Pseudomonas fluorescence* AAUPF 62. These signify that they are compatible (Table 3).

**Table 3:** Compatibility assay among *Trichoderma* and *Pseudomonas* strains

*Trichoderma* strains	Mean mycelial growth (mm) and growth inhibition (%) of *Trichoderma* by *Pseudomonas* strains									
	*Pseudomonas* strains									
	AAUAm28		AAUF3		AAUW 24		AAUS31		AAUPF62	
	Mean ± SD	%	Mean ± SD	%	Mean ± SD	%	Mean ± SD	%	Mean ± SD	%
AAUW1	44.0 ± 1.0	2.2^**−**^	43.7 ± 0.6	3.0^**−**^	45.0 ± 0.0	0.0^**+**^	45.0 ± 0.0	0.0^**+**^	45.0 ± 0.0	0.0^**+**^
AAUC22	45.0 ± 0.0	0.0^**+**^	44.3 ± 1.2	1.5^**−**^	45.0 ± 0.0	0.0^**+**^	45.0 ± 0.0	0.0^**+**^	45.0 ± 0.0	0.0^**+**^
AAUS69	44.0 ± 1.0	2.2^**−**^	45.0 ± 0.0	0.0^**+**^	45.0 ± 0.0	0.0^**+**^	44.0 ± 1.0	2.2^**−**^	44.7 ± 0.6	0.7^**−**^
AAUS12	44.0 ± 1.0	2.2^**−**^	42.0 ± 1.0	6.7^**−**^	45.0 ± 0.0	0.0^**+**^	45.0 ± 0.0	0.0^**+**^	42.0 ± 1.0	6.7^**−**^
AAUA31	44.0 ± 1.0	2.2^**−**^	44.7 ± 0.6	0.7^**−**^	44.3 ± 1.2	1.5^**−**^	44.7 ± 0.6	0.7^**−**^	41.7 ± 1.5	7.4^**−**^
LSD	0.8		2		0.4		0.5		0.6	

*SD* Standard Deviation, compatible (+), incompatible (−)

## Discussion

From this study, it is observed that the faba bean black root rot disease caused by *Fusarium solani* was isolated from infected faba bean roots. The *Fusarium* isolates were identified by their cultural, morphological characteristics, and pot experiment pathogenicity test. White to pale cream colonies was developed after seven days of incubation. Unbranched monophialides with 0–3 septa microconidia and 5–7 septa macroconidia, and chlamydospores were consistent in *Fusarium solani.* The chlamydospores were globose to oval in shape with a smooth or rough wall [30].

Previous research [30, 31] was reported that *Fusarium solani* caused faba bean black root rot disease. In our study, the pathogenicity test confirmed that *Fusarium solani* AAUF51 significantly caused the disease symptoms which was characterized by dark-brown to black discoloration on roots and basal stems under greenhouse conditions. Lower leaves of severely diseased plants turned yellow and blight later. This implies that *Fusarium solani* AAUF51 is the major causal pathogens of faba bean black root and basal stem rot during the vegetative stage in high lands of faba bean growing areas of Ethiopia.

Chemical fungicides have been used for the management of black root rot disease of faba bean. Some researchers, [32, 33] have reported that the efficacy of Mancozeb 80% WP against root rot of plant pathogens. However, chemical fungicides impose hazards to human health and environments [34]. With the rising awareness of the adverse effects of chemical fungicides, people are looking and prefer organically grown crops. Hence, the use of biocontrol agents, play an important role in disease suppression in cultivated plants [19, 35] and could full fill the demand for organic products and sustainable eco-friendly crop management options.

Besides some fungicides, the present study evaluated the in-vitro efficacy of indigenous *Trichoderma* and *Pseudomonas* strains against faba bean black root rot disease-causing *Fusarium solani*. We found the antagonistic efficacy of Mancozeb 80% WP against the test pathogen at 300 and 400 ppm was comparable with the effect of *Trichoderma* and *Pseudomonas* strains. This infers that *Trichoderma* and *pseudomonas* stains are the potential bioagents that can be used to control black root rot disease as a supplementary to chemical fungicide. The application of potential bioagent lonely or in combination with compatible chemical fungicides could minimize the number of fungicides applied to the soil and increase the protection of plants [36].

The in-vitro antagonistic assay of both *Trichoderma* and *Pseudomonas* strains showed that *Trichoderma* AAUW1 exhibited higher mycelial growth percent inhibition (86.67%) of *Fusarium solani* AAUF51 as compared to other antagonistic isolates in dual culture techniques. This could be associated with its mycoparasitism capability. A previous study by [37] demonstrated that *Trichoderma* species usually use multiple biocontrol mechanisms to suppress plant pathogenic fungi. Similarly, [18, 37, 38] have reported the efficacy of rhizosphere *Trichoderma* species against plant pathogens. The *Pseudomonas* AAUPF62 (75.56%) strain showed better antagonism in dual culture against the test pathogen than the remaining bacterial isolates. This study has observed that the antagonistic potential of rhizosphere bacterial isolates that corresponded with the research findings of [26, 39, 40] who have reported that antagonistic activity of *Pseudomonas* species against various plant pathogens. Similarly, [40] has shown that *Pseudomonas aeruginosa* inhibited *Fusarium* isolate by 77.2%. The antagonistic activity of *Pseudomonas* strains against *Fusarium solani* could be attributed to their capacity to produce various antifungal metabolites [30].

The genus *Pseudomonas* is well known of producing large arrays of antifungal metabolites, such as volatile organic compounds [13, 26]. We found the volatile compounds produced by *Pseudomonas aeruginosa* AAUS31 (77.78%) and *Pseudomonas fluorescens* AUUPF62 (71.11%) was more effective in inhibition of the mycelial growth of *Fusarium solani* AAUF51 than other isolates. This could be associated with the antimicrobial properties of volatile metabolites of the bioagents. Besides this, the antagonistic efficacy of the culture filtrates of *Trichoderma* and *Pseudomonas* strains against the test pathogen increased with the concentration of the filtrates. In general, the antagonistic potential of the bioagents could be attributed to the HCN, siderophores, and hydrolytic enzyme production capability of the stains [37, 38]. HCN has been postulated to play an important role in the biological control of pathogens [26, 41]. On the other hand, the hydrolytic enzymes are involved in the cell wall degradation of the fungal pathogens [42].

This study revealed that 11 interactions among *Trichoderma* and *Pseudomonas* strains were compatible with each other as no zone of inhibition was observed between the strains. Our findings were in agreement with [17–19, 43], who reported that, the compatibility of *Pseudomonas and Trichoderma* species under in-vitro assays. In our study, the promising potential antagonistic *Pseudomonas* and *Trichoderma* strains displayed compatibility under in-vitro conditions. *Pseudomonas aeruginosa* AAUS31 and *Pseudomonas fluorescens* AAUPF62 showed a positive interaction with *Trichoderma harzianum* AAUW1 and *Trichoderma viridae* AAUC22. This could be attributed to the existence of synergism between the metabolites produced by the *Pseudomonas* and *Trichoderma* strains*.* The compatible bioagents might protect the pathogens, at different times or under different conditions and might probably mimic the natural situation in the rhizosphere. Generally, it is expected to use that the combined application of compatible antagonists would increase the management of plant pathogens.

## Conclusion and recommendations

This study found *Fusarium solani* as one of the major faba bean distractive fungal pathogen in high lands of faba bean growing areas of Ethiopia. The promising potential antagonistic indigenous *Pseudomonas* (AAUS31 and AAUPF62) and *Trichoderma* (AAUW1 and AAUC22) strains were found compatible with each other under in-vitro conditions. The disease suppression by the combination of compatible bioagents could be more effective than the individual ones possibly by the existence of synergism among the metabolites of the strains. The combined application of compatible *Trichoderma* and *Pseudomonas* strains is recommended for the management of faba bean black root rot disease.

## Data Availability

The data set used/or analyzed during the current study are available from the corresponding author on reasonable request.
